# Immunotherapy: A Challenge of Breast Cancer Treatment

**DOI:** 10.3390/cancers11121822

**Published:** 2019-11-20

**Authors:** Marilina García-Aranda, Maximino Redondo

**Affiliations:** 1 Research Unit, Hospital Costa del Sol, Autovía A-7, km 187, 29603 Marbella, Spain; marilina@hcs.es; 2 Research Network in Health Services in Chronic Diseases (Red de Investigación en Servicios de Salud en Enfermedades Crónicas, REDISSEC), Carlos III Health Institute (Instituto de Salud Carlos III). Av. de Monforte de Lemos, 5. 28029 Madrid, Spain; 3 Malaga Biomedical Research Institute (Instituto de Investigación Biomédica de Málaga, IBIMA), Calle Doctor Miguel Díaz Recio, 28. 29010 Málaga, Spain; 4 Surgery, Biochemistry and Immunology Department, School of Medicine, University of Malaga, 29010 Málaga, Spain

**Keywords:** immunotherapy, breast cancer, resistance, checkpoint, targeted treatment, personalized medicine

## Abstract

Breast cancer is the most commonly diagnosed cancer in women and is a leading cause of cancer death in women worldwide. Despite the significant benefit of the use of conventional chemotherapy and monoclonal antibodies in the prognosis of breast cancer patients and although the recent approval of the anti-PD-L1 antibody atezolizumab in combination with chemotherapy has been a milestone for the treatment of patients with metastatic triple-negative breast cancer, immunologic treatment of breast tumors remains a great challenge. In this review, we summarize current breast cancer classification and standard of care, the main obstacles that hinder the success of immunotherapies in breast cancer patients, as well as different approaches that could be useful to enhance the response of breast tumors to immunotherapies.

## 1. Introduction

### 1.1. Breast Cancer

According to the last Global Cancer Statistics (GLOBOCAN 2018), breast cancer represented 11.6% of all cancers, which places this disease as the second most commonly diagnosed cancer after lung cancer, and caused 6.6% of the total cancer deaths in 2018 [1]. Among women, incidence rates for breast cancer significantly exceeded those for other cancers in both transitioned and transitioning countries, remaining as the most frequently diagnosed cancer and the leading cause of cancer death in women worldwide [1].

Although for the majority of breast cancer patients it is not possible to identify a specific risk factor [2], these are diverse and well documented and include obesity, physical inactivity, alcohol consumption, use of hormone therapy, high breast density, and hereditary susceptibility due to mutations in autosomal dominant genes [3], which represents between 5–10% of all breast cancer cases in women [3]. Among these genetic alterations, mutations affecting *BRCA1* and *BRCA2* genes, which control DNA repair and transcriptional regulation in response to DNA damage, can lead to the accumulation of genetic alterations and greatly increase lifetime risk to develop different types of malignancies, including breast cancer [4]. Indeed, mutations in BRCA1 and BRCA2 genes are associated with an increased risk of inherited breast and ovarian cancer, representing the strongest susceptibility markers that have been identified for breast cancer worldwide, with an estimated 45–80% lifetime risk of breast cancer for BRCA1-BRCA2 mutation carriers [4]. In a similar manner, mutations affecting *TP53* are also related to triple negative breast cancer [3].

As with other types of cancer, early diagnosis greatly increases the chances for successful treatment, allowing for a 20% reduction in overall mortality rates [5]. In this regard, despite reported handicaps of screening programs like high overdiagnosis rates and costs, risks that are derived from ionizing radiation, or false positive biopsy recommendations, both mammography, breast self-examinations, clinical breast examinations, digital breast tomosynthesis, ultrasonography, magnetic resonance imaging, and oncogene identification represent the main tools for early diagnosis, sorting out, and prevention of risk factors as well as timely treatment to lessen breast cancer morbidity [5].

Besides screening programs, adjuvant chemotherapy has also had a significant impact on the prognosis of breast cancer patients, having significantly improved their overall survival, disease-free survival [6], and death rates related to breast-cancer since the early 1990s [7]. In this respect, breast cancer has traditionally been classified into three subtypes with different prognoses and treatment responses [8,9] (Table 1).

Even though most breast cancer patients are diagnosed early enough to be successfully treated with surgery, chemotherapy, radiotherapy, or a combination thereof [8], nearly 30% of women that are initially diagnosed with early-stage disease will eventually develop a metastatic disease [7], which ultimately leads to patient death. In this scenario, and given their high efficacy and selectivity, the development of cancer immunotherapies and other treatment strategies targeting tumor cells have positioned themselves as promising options to win the battle against breast cancer in opposition to conventional treatments that lack tumor selectivity and cause more side effects.

### 1.2. Immunotherapy as an Option for Cancer Treatment

**Figure 1:**
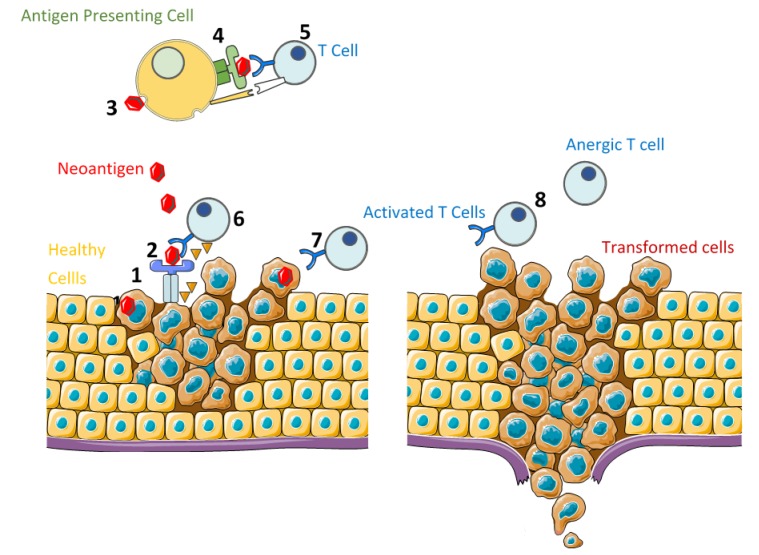
Cancer Immunoediting. ELIMINATION: (1) Neoantigen production by transformed cells. (2) Neoantigen presentation on the surface of transformed cells, associated with HLA-I. (3) Neoantigen recognition and processing by antigen presenting cells. (4) Neoantigen presentation on the surface of antigen presenting cells, associated with HLA-II. (5) T-cell activation in the presence of co-stimulatory signals. (6) Transformed cell recognition by activated T cells and elimination. EQUILIBRIUM: (7) Transformed cells with a resistant or non-immunogenic phenotype escape elimination and proliferate, although the immune system is still able to control the tumor growth. ESCAPE: (8) Uncontrolled proliferation of cells with a resistant or a non-immunogenic phenotype, leading to tumor progression and metastasis.

According to the cancer immunoediting model [15], the relation between tumor cells and the immune system is a dynamic process which consists of three main phases (Figure 1):

1. Elimination: During this phase, cancer cells are successfully recognized and destroyed by the body’s immune system [16]. The success of the immune system to eliminate tumor cells depends on the ability of the antigen to trigger the immune response, or immunogenicity, which can be summarized as follows:
  - Genetic abnormalities lead to the production of new antigens by tumor cells, which are processed and presented as antigen-derived peptides on the cell surface in association with Human Leukocyte Antigen class I (HLA-I).
  - Neoantigens that are present in tumor microenvironment are recognized, processed, and presented on the surface of Antigen Presenting Cells (APCs) as antigen-derived peptides in association with Human Leukocyte Antigen class-II (HLA-II), which can be recognized by helper T-cell receptors and leads to B-cell and cytotoxic T-cell stimulation and maturation.
  - After T-cell activation by co-stimulatory signals provided by APCs, T-cells recognize neoantigens presented by HLA-I and attack the targeted tumor cell by the secretion of cytotoxic granules and/or via Fas cell surface death receptor (FAS) and caspase activation.
2. Equilibrium: During this phase, transformed cells with a resistant or non-immunogenic phenotype escape the elimination phase and proliferate, although the immune system is able to control the tumor growth [16].
3. Escape: The selective pressure caused by anti-cancer treatments or immune-surveillance promotes the uncontrolled proliferation of cells with a resistant or a non-immunogenic phenotype, leading to tumor progression and metastasis.

One of the characteristics of advanced tumors is their capability to evade adaptive immune responses [17], which would explain the direct relationship between tumor growth and immune evasion [18]. Since mechanisms leading to tumor evasion are diverse [19] (Table 2), a significant effort has been made in recent years to develop new strategies to trigger tumor cell death by stimulating the patient’s natural defenses to recognize and destroy tumor cells.

**Figure 2:**
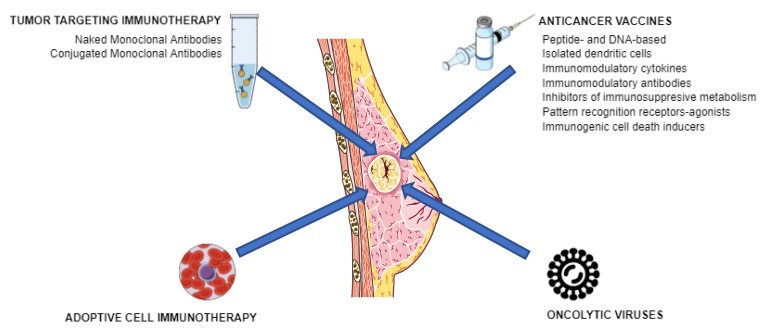
Modalities of cancer immunotherapy.

These findings have been the basis for the development of different modalities of anticancer immunotherapy, including tumor-targeting immunotherapies, oncolytic viruses, anticancer vaccines, or adoptive cell immunotherapies [24] that are designed to target tumor cells and work with the immune system at different levels (Figure 2). As a result of the great success achieved in different studies and trials that demonstrate the efficacy of immunotherapies not only against primary tumors, but also preventing metastasis and recurrence [25], cancer immunotherapy has become the fourth pillar of cancer care, complementing surgery, cytotoxic therapy, and radiotherapy [26].

In this regard, the recent approval of treatments based on the use of checkpoint inhibitors has been a turning point for the treatment of patients with different tumors including melanoma, non-small cell lung cancer, renal cell carcinoma, Hodgkin’s lymphoma, bladder cancer, head and neck squamous cell carcinoma, Merkel-cell carcinoma, microsatellite instability high, or mismatch repair deficient solid tumors [27], since they have demonstrated that they significantly increase survival rates when compared to standard therapy among different tumor types [23]. The impact of the basic studies allowed the development of checkpoint inhibitor therapies, which is the reason that James P. Allison and Tasuku Honjo won the Nobel Prize in Physiology and Medicine in 2018.

### 1.3. Checkpoint Inhibitors

Provided that activated CD8^+^ (cytotoxic T lymphocytes or T-cells) recognize and destroy pathogen-infected or aberrant cells like cancer cells, they are considered the main effectors of cell-mediated immunity. On the other hand, since T cells also increase antibody responses through the action of CD4 (T-helper cells) and the enhancement of antibody production by B cells, their activation represents a critical step for the initiation and regulation of the immune response [28].

In accordance with the two-signal model of lymphocyte activation, both co-stimulatory signals and antigen-specific signals mediating the engagement of T-cell receptor (TCR) to HLA-II expressed on the surface of antigen-presenting cells participate in T-cell activation and maturation [29]. The subsequent response is regulated by a balance between co-stimulatory and inhibitory signals, or immune-checkpoints [30], at multiple steps during the immune response [31], which limits tissue damage and allows for the maintenance of self-tolerance. Given their immunosuppressive functions, dysregulated expression of inhibitory signals implies a major advantage in the tumor microenvironment, leading to immune evasion (Table 2).

Nowadays, and due to their association with the inhibition of lymphocyte activity and subsequent anergy, different immune checkpoint receptor-ligand combinations are the subject of intense study as tools for cancer treatment by restoring immune system function either as mono or in combination therapies [30,32]. Among these, and because of their central role during the immune response and peripheral tolerance, both Cytotoxic T-Lymphocyte-Associated Antigen 4 (CTLA4, CD152) and Programmed Cell Death Protein 1 (PD-1, CD279)/Programmed Cell Death Protein 1 Ligand (PD-L1, CD274) pathways have proved to be valid targets for the development of new cancer treatments and have allowed for the clinical approval of a number of CTLA and PD-1/PD-L1 checkpoint inhibitors (Table 3).

As previously mentioned, apart from T-cell receptor interaction with HLA-II, T-cell activation is controlled by further antigen-independent costimulatory signals such as CD28 (Cluster of Differentiation 28) and CTLA-4. In this respect, and contrary to CD28 signals, which are required for T-cell activation and cytokine secretion, CTLA-4 signaling inhibits T-cell activation, which is especially important in lymph nodes where CTLA4 neutralizes potentially autoreactive T-cells at the initial stage of naïve CD4 and CD8 cell activation [31]. Both CD28 and CTLA-4 can be stimulated by CD80 (B7-1) and CD86 (B7-2) ligands that are expressed on activated APCs, leading to T-cell proliferation and differentiation through the production of growth cytokines when there is an elevated CD28:CD80/CD86 ratio [42] or to dephosphorylation of T-cell receptor signaling proteins by tyrosine phosphatases [43], leading to T-cell inactivation and anergy, in the case of an increased CTLA-4:CD80/CD86 ratio [42].

Since CTLA-4 binds to CD80/86 with very high affinity, this receptor mediates immunosuppression by competing for CD28 and also by inducing CD80/86 removal from antigen presenting cells’ surface [33]. For this reason, by blocking the interaction between CTLA-4 and CD80/86 ligands, CTLA-4 inhibitors can prevent T-cells exhaustion and boost the antitumor T-cell response [44]. Despite the demonstrated survival benefit of ipilimubab in patients with advanced melanoma, severe immune-mediated adverse effects, high cost, and modest response rates (ranging between 4% and 16% when used in monotherapy) [44] remain as the main impediments for its use.

On the other hand, PD-1 predominantly regulates previously activated T-cells at the later stages of an immune response [31], mainly within tissue and tumors [30,31]. The expression of this membrane receptor, which can be temporarily induced in activated CD8 T-cells, natural killer T-cells, or myeloid cells following the activation and stimulation of T-cell receptor by cytokines and interleukin, is constitutive in T-cells exhibiting the exhausted phenotype [23]. PD-1 binding to its ligand, PD-L1, promotes the dephosphorylation of T-cell receptor proximal signal components and leads to the inhibition of signaling pathways commanded by protein kinases including PI3K/AKT (phosphoinositide 3-kinase/Protein Kinase B), PTEN (phosphatase and tensin homolog), CK2 (casein kinase 2) [37], and RAS/MEK/ERK (mitogen-activated protein kinase MAPK/extracellular-signal-regulated-kinase), which decreases T-cell proliferation, survival, cytokine production, and other effector functions [23]. Thus, by interrupting the interaction between PD-1 and PD-L1, checkpoint inhibitors can restore antitumor immune responses and promote immune-mediated elimination of tumor cells [32]. Although nivolumab alone or combined with ipilimumab significantly improves the overall and complete response rates compared with ipilimumab alone in patients with metastatic melanoma [45], response rates to PD-1/PD-L1 blocking therapies only ranges between 20–38% among different tumor types [23], which implies that the majority of advanced stage patients cannot benefit from these therapies.

Despite the low response rates and immune-related adverse events in some cancer patients, both CTLA4 and PD-1/PD-L1 inhibitors have broadly demonstrated their value to boost potent and durable anti-tumor responses and to increase the average life expectancy for metastatic cancer patients [23].

## 2. Breast Cancer Immunotherapy

### 2.1. First Approaches

The first cancer immunotherapy treatments were based on the use of humanized monoclonal antibodies with the ability to bind and neutralize a targeted altered molecule expressed by cancer cells and on which their survival and proliferation depends. The approval in September 1998 of trastuzumab (Herceptin^®^, Genentech, Inc., South San Francisco, CA, United States) represented the release of the first antibody for the treatment of metastatic breast cancer patients with HER2 (Receptor tyrosine-protein kinase ERBB2, CD340) overexpression and/or gene amplification, which represented a milestone in the treatment of breast cancer. After trastuzumab, other different anti-HER2 monoclonal antibodies including lapatinib (Tykerb^®^, GlaxoSmithKline, Brentford, United Kingdom), neratinib (Nerlynx^®^, Puma Biotechnology, Los Angeles, CA, United States), gefitinib (Iressa^®^, AstraZeneca, Cambridge, United Kingdom), or afatinib (Giotrif^®^, Boehringer Ingelheim Pharmaceuticals, Inc., Ingelheim am Rhein, Germany) [8] as monotherapy or in combination with conventional treatments have contributed to increasing the number of therapeutic options for breast cancer patients.

Although the use of monoclonal antibodies targeting altered proteins has definitely improved the outcome of cancer patients, modest response rates (Table 4) and resistance development [46] remain as the major impediments for treatment success and require the search for new approaches apart from combined therapies, among which antibody-drug conjugates (ADC) such as the recently FDA approved ado-trastuzumab emtansine (Kadcyla^®^, Genentech, Inc., South San Francisco, CA, United States) [47] and T cell bispecific antibodies stand out among the most promising strategies for breast cancer patients [48].

As a result of the latest studies in this field and in line with the encouraging long-term success of checkpoint inhibitors in the treatment of different tumors, distinct research groups have focused their efforts in developing analog treatments for breast cancer patients. In fact, as a result of the findings from the Phase III double-blind IMpassion130 trial (ClinicalTrials.gov ID NCT02425891), which reported a 40% reduced risk of disease progression or death in patients receiving atezolizumab plus nab-placlitaxel or placebo [58,59,60], in March 2019, the FDA approved the first checkpoint inhibitor immunotherapy drug, the anti-PD-L1 antibody atezolizumab (Tecentriq ^®^), in combination with chemotherapy (Abraxane^®^) for the treatment of triple-negative, metastatic breast cancer patients with positive PD-L1 protein expression [61]. However, despite this great milestone, modest complete response rates (7.1%, 95% CI, 4.9–9.9 and 10.3%, 95% CI, 6.3–15.6 in PD-L1 positive subgroup) and immune-mediated serious adverse events such as pneumonitis, hepatitis, colitis, and endocrinopathies that can cause treatment discontinuation [59] remain as notable impediments for the success of this treatment and justify the search of new therapeutic strategies.

### 2.2. Mechanisms of Immune Evasion in Breast Cancer

As stated above, tumor immune evasion can occur as a result of defective tumor-directed T-cell activation, deficient activated T-cell infiltration into the tumor microenvironment, or because of the tumor cell resistance to cytotoxic action of the immune cells [62].

#### 2.2.1. Breast Tumor Microenvironment

Immunogenicity is defined as the ability to induce a humoral and/or cell-mediated adaptive immune response. In fact, both the burden of tumor mutations and the load of neo-epitopes represent two of the factors that are linked to response to checkpoint inhibitors in different malignancies like melanoma or lung cancer [63]. However, although tumor neoantigens that are produced as a result of breast cancer cells’ genomic instability can be recognized by the immune system and induce T-cell responses and antitumor immunity [62,64], the immunogenicity of breast cancer can be rather heterogeneous, depending to a large extent on the specific subtype of breast cancer [65].

In the particular case of HER2-positive breast tumors, gene profiling studies have shown that highly suspicious calcifications are associated with decreased immune system activity and *ERBB2* overexpression [66]. For this reason, breast calcifications would be useful not only in the radiological assessment of breast lesions [67], but also in the management of breast cancer patient candidates for immunotherapy. On the other hand, although estrogen receptor-negative and HER2-positive have shown evidence of immunogenicity [65], these types of inflammatory breast tumors are rare (1–5% of cases) [68] when compared to triple negative breast tumors, which are unique among breast cancer subtypes in having strong antigen expression [69] and high stromal and tumor-infiltrating lymphocytes, parameters with a strong prognostic and predictive significance to immunotherapy and chemotherapy [62,63,70,71]. Accordingly, triple negative breast tumors with high infiltration of tumor-associated macrophages have been found to have a higher risk of metastasis and lower rates of disease-free survival and overall survival, having been proposed as potentially useful prognostic markers for triple negative breast cancer patients [72,73].

Except for these immunogenic subtypes, breast tumors have historically been classified as immunologically silent [62] or “cold” tumors, characterized by the presence of low mutation and neoantigen burden and few effector tumor infiltrating lymphocytes, factors proposed as prognostic markers [62], and metastasis to lymph nodes correlation [74].

Since non-inflamed tumors represent a significant impediment to the success of T-cell-based immunotherapies, different studies have aimed their efforts towards developing new strategies to increase the presence of immune infiltrates and hence, to improve patient prognosis. Among these, in addition to directly causing cell damage [75], the use of local tumor hyperthermia has proven to be a valuable tool as an immunotherapy strategy for cancer [76] by boosting immune cell activation and increasing the sensitivity of tumor cells to anti-tumor immune responses by different mechanisms, including:

- Enhancing the expression of tumor surface HLA class I polypeptide-related sequence A (MICA) and HLA type I, which promote tumor cell sensitivity to lysis by NK cells and CD8^+^ cells, respectively [75].
- Increasing the release of heat shock proteins, which leads to NK cells activation as well as to APCs activation and antigen presentation to CD8^+^ cells [75].
- Increasing the release of tumor cells exosomes, which apart from containing chemokines, transfer potential tumor antigens to APCs and subsequent CD8^+^ activation [75].
- Promoting changes in the tumor vasculature, which facilitates better trafficking of immune cells between the tumor and draining lymph nodes [75].

In this context, different studies are reporting promising results for hyperthermia as complementary treatment to surgery, chemo, radio, and immunotherapy in breast cancer patients [75,77,78,79]. However, convincing data about the benefit of the combination of hyperthermia with checkpoint inhibitors for breast cancer treatment should be provided by multicenter clinical trials in which related side-effects are also evaluated [79]. Likewise, radiation has also shown to increase mutational load of tumors, optimize antigen presentation, and decrease immune suppressors in the tumor microenvironment, priming the tumor for immunotherapy [71], which justifies additional studies in these fields.

Besides the presence of high tumor infiltrating lymphocytes, recognition of tumor cells is a critical step for the success of the immune response. In this regard, although estrogen has an immunoenhancing impact on the immune system [80] with an apparent effect in all major innate and adaptive immune cells [81], high levels of estrogens may interfere with HLA-II expression and IFN-γ signaling, with significant implications for tumor immune escape [82]. Estrogens are also well known to be a risk factor for breast cancer by enhancing the expression of genes involved in tumor cell survival and proliferation as well as growth factors including vascular endothelial growth factor (VEGF) [83], epidermal growth factor (EGF), insulin growth factor (IGF), fibroblast growth factor (FGF) [69,84], and their receptors [8]. Since estrogen presence in tumor microenvironment can also play a significant immunosuppressive role by promoting tolerance of weakly immunogenic tumor cells [69], the use of antiestrogen therapies in combination with aromatase inhibitors could be a rational strategy to enhance the response to immunotherapies. However, although adjuvant hormonal therapy combined with HER2-targeted agents in hormone receptor-positive and HER2-positive breast cancer patients already represents a standard treatment, recent studies have shown that estrogen deprivation promotes transcriptional programs that favor immune evasion and increases PD-L1 expression in metastasis arising from breast cancer patients receiving adjuvant hormonal therapy for their local disease [85]. For this reason, the use of hormone-therapies in combination with PD-1/PD-L1 blocking immunotherapies should be thoroughly investigated. On the other hand, and for the reasons mentioned above, the application of conventional monoclonal antibodies targeting one or more growth factors would be a useful adjuvant to enhance the efficacy of breast cancer immunotherapy by improving APCs function [86,87].

#### 2.2.2. Changes in Breast Tumor Cells

Instead of loss of the targeted protein, resistance to cancer immunotherapies, such as monoclonal antibodies, is frequently due to the activation of alternate pathways [53] like immunosuppressive checkpoint pathways.

Among these, and largely due to the FDA approval of atezolizumab, blockade of the PD-1/PD-L1 pathway constitutes one of the most promising strategies for breast cancer immunotherapy. Despite this important addition to the number of therapeutic options available for metastatic breast cancer patients, it is important to note that the objective response rate achieved by atezolizumab was 53% versus 33% for the placebo group [88] and that to date, it has been approved for the treatment of the triple-negative subtype, which only constitutes 10–15% of breast carcinomas [89], with positive PD-L1 protein expression, which occurs in approximately 20% of breast cancers (mainly HER2-positive and triple negative) [62].

Similarly to the PD-L1 pathway, different randomized clinical trials are currently evaluating the effect of PD-1 inhibitors as monotherapy or in combination with conventional and non-conventional treatments [62,65] in breast cancer patients with results that although modest, are encouraging. In this respect, even though PD-L1 status remains the core predictor for anti-PD-1/PD-L1 therapies and patient selection [23], the validity of PD-L1 expression as a prognostic marker remains controversial [62] and justifies the need to develop new immunotherapy biomarker panels as well as new strategies to improve response rates.

Another major impediment to immunotherapy success in breast cancer patients is the selection of apoptosis-resistant cells, which constitutes one of the hallmarks of cancer [17]. Since both chemo- and immunotherapies directly or indirectly activate the cellular apoptosis machinery, tumor sensitivity to anti-cancer treatments will significantly depend on the level of expression of anti-apoptotic proteins [24] in general and, more specifically, on the existence of a pro-survival profile characterized by an increased ratio between anti-/pro-apoptotic proteins [24,90,91].

Provided that antiapoptotic proteins such as clusterin (APO-J) [91,92], BCL-2, BMF [24] as well as different pro-survival kinases [8] are frequently altered in metastatic breast cancer, the use of profiling techniques or systematic mapping of anti-apoptotic gene dependencies would be justified in order to effectively select those patients that could better benefit from combined treatments of protein inhibitors and immunotherapy. In this regard, different studies have already evidenced the need to use therapies with a combination of inhibitors targeting different anti-apoptotic proteins in order to achieve better clinical benefits and avoid the activation of alternate pro-survival pathways [8,23,24].

HLA-I expression on the surface of breast tumor cells, which is positively correlated with tumor-infiltrating lymphocytes, is essential for an effective cytotoxic response [93] and the subsequent success of T-cell mediated immunotherapies. For this reason, loss or changes in HLA-I expression, which is another of the hallmarks of cancer [17], also represent a significant impediment for breast cancer immunotherapy.

Total loss of HLA-I is found in 37% of in situ breast carcinomas, 43% of the primary tumors, and 70% of the lymph node metastases [94]. Since HLA-I expression in these tumors is related with a pro-death phenotype characterized by an increased proapoptotic *BAX*/antiapoptotic *BCL2* ratio [94], preliminary studies for patients’ selection would be justified in order to ensure the success of immunotherapies in breast cancer patients.

In the case of triple negative breast tumors, HLA-I expression is variable, contributing when altered to the development of an immunosuppressive tumor microenvironment and immune escape [69]. However, since the activation of the HLA-II presentation pathway occurs in approximately 30% of triple negative breast cancer patients [95], associated with the presence of tumor infiltrating lymphocytes and improved prognosis [95,96], the expression of both receptors are factors that must be taken into consideration prior to the application of immunotherapy treatment.

With respect to HER2 overexpressing tumors, although this receptor-tyrosine kinase represents a valuable target for T-cell based immunotherapies, these tumors may escape cytotoxic T lymphocyte-mediated lysis by downregulating HLA-I, since the expression of both receptors is inversely correlated with breast cancer cells [69,97]. Similarly, in normal and cancerous breast tissues, HLA-I expression is inversely correlated with the expression of estrogen receptors, which may be related to the low level of tumor-infiltrating lymphocytes [93], and hence, with the failure of the T-cell cytotoxic response. It is worth emphasizing at this point that provided that agents targeting different protein kinases such as Mitogen Activated Protein Kinase (MAPK) or HER2 may increase HLA-I expression in breast cancer cells [97,98], the use of kinase inhibitors would be a valuable strategy to increase the antitumor effects of T-cell based immunotherapies. Similarly, strategies aimed at inducing HLA-II expression in tumor cells may be valuable tools to increase patient response and prognosis to such therapies [96].

## 3. Conclusions

### Where to go

The improvement of the response rates for immunotherapies remains a great challenge for cancer treatment in general and for breast cancer in particular. Considering the relatively limited T-cell infiltration in most breast cancers, the development of novel strategies that are aimed to enable sufficient lymphocyte infiltration as well as to generate de novo T-cell responses that overlap the immunosuppressive tumor environment may be key to the success of this kind of therapy in breast cancer patients [63].

Among the different approaches that are currently being considered and despite their limited efficacy when delivered as a monotherapy [99], oncolytic viruses have demonstrated their safety [100] and ability in targeting and killing cancer cells as well as in stimulating immunotherapeutic effects in patients [101], positioning themselves as a promising strategy to increase treatment efficacy when used in combination therapy [99,100,101] and as a unique platform for personalized treatment of patients with advanced breast cancer [101].

Recent evidence on the role of tumor-associated macrophages in breast tumor growth, progression, treatment resistance, and metastasis has paved the way for the development of novel macrophage-targeted breast treatment strategies, such as the inhibition of macrophage recruitment, repolarization of tumor-associated macrophages to an antitumor phenotype, and the enhancement of macrophage-mediated tumor cell killing or phagocytosis, which are currently being evaluated in clinical trials [102]. Despite the promising results of preclinical studies, these therapies have proved limited clinical efficacy, therefore the development of new strategies that improve the effectiveness of such treatments is necessary.

On the other hand, results of adoptive cell immunotherapies, which includes Chimeric Antigen Receptor (CAR) T cell therapy and Tumor-Infiltrating Lymphocyte (TIL) therapy, based on the isolation of antitumor T cells from the primary tumor, further ex vivo expansion and activation, and subsequent reinfusion of such cells into the patient [65], are also proving valuable in both preclinical and clinical studies for the treatment of patients with breast cancer in general and HER2 positive tumors in particular [65,103]. In a similar line, next-generation sequencing and bioinformatic technologies have become a fundamental tool to facilitate neoantigen identification and the consequent improvement of personalized neoantigen-based translational immunotherapy studies [104], as well as to develop neoantigen vaccines to induce neoantigen-specific T-cell responses through the activation of antigen-presenting cells [105,106].

Results obtained with nanoparticles are no less important, having been postulated as the great asset to overcome the limitations of existing immunotherapy, being able to improve overall anti-cancer immune responses with minimal systemic side effects [107]. However, although nanoparticles in different in vitro and in vivo breast cancer models [108] have already proven their efficacy in defeating the immune-suppressive effect of tumor microenvironment [107] and drug resistance [108], as well as in delivering neoantigens and adjuvants to tumor cells [107], reducing the side effects of anticancer drugs [109], certain nanoparticles like titanium dioxide, silica, and gold complexes can lead to the formation of micrometer-size gaps in the blood vessel’s endothelial walls and the intravasation of surviving cancer cells into the surrounding vasculature, which increases the risk of metastasis [110].

Another main drawback of immunotherapies, especially within a combined regimen, is the occurrence of immune-related side-effects affecting different organs such as the skin (rash, pruritus) or gastrointestinal tract (diarrhea, colitis) (Table 4). Although the severity of these immune-related adverse events are generally mild, life-threatening complications may also occur [111] and would end up, in many cases, in a reduction of the optimal treatment dose or medication discontinuation. For this reason, there is still a strong need for further research in order to develop biomarker panels that allow for patient selection and predict the response to immunotherapies and immune-related adverse events.

## Figures and Tables

**Table 1:** Breast cancer classification and standard of care.

Subtype	Overview	Standard of Care
HR+: Luminal-A, Luminal-B	This subtype accounts for up to 75% of breast cancer tumor cases [10] and is characterized by being hormone receptor positive. Luminal A breast tumors, which represent 50–60% of all breast cancers, are defined as ER+ and/or PR+, HER2-, and low Ki67 (<14%) [9,10]. These tumors usually exhibit low histological grade, low mitotic activity, and good prognosis [10]. Luminal B tumors, which represent 15–20% of breast cancers, are defined as ER+ and/or PR+/- (PR<20% + Ki67≥14%) with HER2- as well as ER+ and/or PR+/- (any PR+ and any Ki67) and HER2+ [9,11]. These tumors are generally characterized by a more aggressive phenotype with a higher histological grade and proliferative index than Luminal A tumors [10]. Indeed, although Luminal B tumors respond better to neoadjuvant chemotherapy, they usually present worse prognoses [10].	Sensitive to hormone-targeted treatments, with a response rate of approximately 50–60%. Tamoxifen (TMX, Novaldex^®^) and aromatase inhibitors are the most common drugs that are used in clinical practice as first-line treatments. However, natural or acquired resistance to treatment along with long-term toxicities limit the effectiveness of the treatment [8].
HER2-Enriched	Constitutively activated in 20–30% of breast cancers, being responsible for dysregulated cell proliferation [12] and aggressive biological and clinical behavior [10]. These tumors are defined as ER-, PR-, and HER2+ [11].	Humanized monoclonal antibodies against HER2 extracellular domain and small kinase inhibitors [8]. Acquired resistance to treatment is a recurrent problem for HER2-enriched breast cancer patients.
Basal-Like	TNBC tumors, which constitute approximately 80% of the basal-like tumors and account for 10–15% of breast carcinomas [8], are defined as ER-, PR-, HER2-, CK5/6+, and/or EGFR+ [11].	Chemotherapy is the current standard of care for advanced TNBC despite limited efficacy and poor survival outcomes [13]. Different targeted treatments for TNBC are under pre-clinical or clinical development [13,14].

HR+: Positive for Hormone Receptors. ER+: Expressing Estrogen Receptors. PR+: Expressing Progesterone Receptors. HER2: Positive for Human Epidermal Growth Factor Receptor 2 (Receptor tyrosine-protein kinase ERBB2, CD340). TNBC: Triple Negative Breast Cancer. CK5/6+: Expressing cytokeratin 5/6. EGFR+: Expressing Epidermal Growth Factor Receptor.

**Table 2:** Immune system mechanisms of tumor evasion.

Target	Mechanism	Overview
Alterations in APCs	Inhibition of APC maturation and activation which impedes the appropriate co-stimulatory and cytokine signals to T cells and triggers the generation of regulatory T cells [20].	Different factors present in the tumor microenvironment such as IL-6, M-CSF, IL-10, VEGF, and TGF-β negatively regulate antigen-presenting cell functions [21].
	Selective increase in regulatory APCs that prevent immune responses by secreting TGF-β and stimulating the proliferation of regulatory T-cells [20].	Tumor microenvironment can induce a selective increase in the number of regulatory APCs, which can induce T-cell unresponsiveness by controlling T-cell polarity [20].
Dysfunction of effector cells	Enhanced proliferation of regulatory T-cells that suppress inflammation and regulate immune system activity.	Tumor microenvironment induces the proliferation of regulatory T-cells, which are able to inhibit T-cell proliferation and cytokine production, leading to immune suppression, which favors the immune escape of tumor cells [20].
	Induction of effector T-cells apoptosis through tumor-generated CD95L and activation of the T-cell CD95 receptor.	CD95 and CD95L are critical survival factors for cancer cells that protect and promote cancer stem cells [22]. Apart from suppressing the immune response, CD95L promotes tumor growth and invasiveness and triggers the acquisition of cancer stem cell phenotypes [22].
	Alterations in T-cell signal transduction after antigen stimulation which leads to a decreased response.	Alterations such as the decreased expression of CD3ζ, p56^lck^, and JAK-3, decreased mobilization of calcium signaling, inability to translocate NF-ĸB-p65, or decreased production of IL2 are frequently found in cancer patients [19].
Changes in tumor cells	Selection of tumor cells that are resistant to apoptosis, one of the hallmarks of cancer [17].	The pressure of immune surveillance or chemotherapeutic drugs enhances the selection and proliferation of cancer cells with mutations or alterations affecting one or various pathways controlling apoptosis.
	Alterations in HLA I expression.	Since the initiation of adaptive immune response occurs after T-cell receptor binding to antigen-loaded HLA-I presented by tumor cells, alterations in HLA-I expression, which is found in approximately 40–90% of human tumors derived from HLA-I positive tissues [23], impedes T-cell activation or causes loss of recognition.
	Alterations in the immune checkpoints.	After recognition of peptide antigen associated with the HLA-I, T-cell activation is controlled by co-stimulatory and co-inhibitory receptors and their ligands (immune-checkpoints). The over-expression of co-inhibitory molecules or the absence of co-stimulatory molecules typically leads to a T-cell exhausted phenotype.

CD95: Fas/APO-1. CD95L: CD95 ligand. APC: Antigen Presenting Cell/Dendritic Cell. HLA: Human Leukocyte Antigen. IL: Interleukin. JAK-3: Janus kinase 3. M-CSF: Macrophage Colony-Stimulating Factor. NF-ĸB: Nuclear Factor-kappa-B transcription complex. TGF: Transforming Growth Factor. VEGF: Vascular Endothelial Growth Factor.

**Table 3:** Approved checkpoint inhibitors for cancer treatment.

Immune Checkpoint Target	Overview	Approved Drugs
CTLA4 (CD152)	One of the co-inhibitory proteins constitutively expressed on the surface of regulatory T cells (Tregs) and frequently upregulated in other types of T cells, like CD4^+^ T, cells upon activation, and exhausted T cells, among other inhibitory receptors [33]. CTLA-4 blockade prevents interaction with CD80/86 resulting in up-regulation of T-cell activity.	Yervoy ^®^ (ipilimumab, Bristol Myers Squibb) was first approved by the FDA in 2011 and is classified as monotherapy for the treatment of advanced melanoma [34]. In combination with nivolumab (Opdivo^®^), ipilimumab is also classified as a first-line treatment for adult patients with intermediate/poor-risk advanced renal cell carcinoma, patients with nonresectable or metastatic melanoma across BRAF status, and previously treated MSI-H or dMMR metastatic colorectal cancer [35].
PD-1 (CD279)	PD-1 is one of the co-inhibitory membrane receptors of which its expression can be induced in active T cells upon stimulation of T-cell receptor complex or exposition to different cytokines [33]. Since PD-1 binding to its ligands, PD-L1 and PD-L2, leads to T-cell inactivation, PD-1 blockade enhances T cell-mediated immune responses.	Opdivo^®^ (nivolumab, Bristol-Myers Squibb) is a PD-1 blocking antibody that, after first being approved by the FDA in 2014, is recommended for the treatment of advanced melanoma, advanced non-small cell lung cancer, advanced small cell lung cancer, advanced renal cell carcinoma, classical Hodgkin lymphoma, advanced squamous cell carcinoma of the head and neck, urothelial carcinoma, MSI-H or dMMR metastatic colorectal cancer, and hepatocellular carcinoma [36]. A combined regimen with ipilumubab increases progression-free survival and overall survival only in patients with low tumor PD-L1 expression [37].
		Keytruda^®^ (pembrolizumab, Merck KGaA) is a human PD-1-blocking antibody that was first approved by the FDA in 2014 and is recommended for the treatment of advanced melanoma, non-small cell lung cancer, head and neck cancer squamous cell carcinoma, classical Hodgkin lymphoma, primary mediastinal large B-cell lymphoma, urothelial carcinoma, MSI-H cancer, gastric cancer, cervical cancer, hepatocellular carcinoma, Merkel cell carcinoma, and renal cell carcinoma [38].
		Libtayo^®^ (cemiplimab-rwlc, Sanofi S.A.) is a PD-1 blocking antibody that was first approved by the FDA in 2018 and is indicated for the treatment of patients with metastatic cutaneous squamous cell carcinoma [39].
PD-L1 (CD274)	One of the immune inhibitory receptor ligands expressed by hematopoietic, non-hematopoietic cells such as T-cells and B-cells and different types of tumor cells.	Tecentriq^®^ (atezolizumab, Genentech Inc.) is a PD-L1 blocking antibody that was first approved by the FDA in 2016 and is recommended for the treatment of advanced urothelial carcinoma, metastatic non-small cell lung cancer, and extensive-stage small cell lung cancer for use in combination with Abraxane^®^ for the treatment of metastatic triple-negative breast cancer [38].
		Bavencio^®^ (avelumab, Merck EMD Serono) is a PD-L1 blocking antibody that was first approved by the FDA in 2017 and is used for the treatment of patients with metastatic Merkel cell carcinoma, advanced or metastatic urothelial carcinoma, and in combination with axitinib for patients with advanced renal cell carcinoma [40].
		Imfizi^®^ (durvalumab, AstraZeneca plc) is an anti PD-L1 human monoclonal antibody that was first approved by the FDA in 2017 and is used for the treatment of patients with unresectable non-small cell lung cancer that has not progressed after chemoradiation [41].

CTLA4: Cytotoxic T-lymphocyte associated protein 4; PD-1: Programmed Cell Death Protein 1; PD-L1: Programmed Cell Death Protein 1-Ligand; MSI-H: Microsatellite instability high; dMMR: Mismatch repair deficient.

**Table 4:** Approved humanized monoclonal antibodies for breast cancer treatment.

Monoclonal Antibody	Response Rates (Monotherapy)	Most Common Treatment-Related Adverse Events
Trastuzumab	35% (95% CI, 24.4% to 44.7%) and none in patients with 3+ and 2+ HER2 overexpression by immunohistochemistry, respectively [49]. Further, 34% (95% CI, 23.9% to 45.7%) and 7% (95% CI, 0.8% to 22.8%) in patients with and without HER2 gene amplification by fluorescence in situ hybridization analysis, respectively [49]. Approximately 15% of patients relapse after therapy [50].	Chills (25%), asthenia (23%), fever (22%), pain (18%), nausea (14%), cardiac dysfunction (2%) [49].
Pertuzumab	3% to 7.6% complete response and 16.7% partial response in previously trastuzumab-treated breast cancer patients [51,52].	Diarrhea (48.3%), Nausea (34.5%), vomiting (24%), fatigue (17%), asthenia (17%), back pain (10%) [51].
Lapatinib	24% in trastuzumab-naïve and less than 10% in trastuzumab-refractory breast tumors [53]. Partial response in 39% (95% CI, 30% to 48%) of patients with relapsed or refractory HER2-positive inflammatory breast cancer [54].	Diarrhea (59%), fatigue (20%), nausea (20%), rash (18%), anorexia (16%), dyspnoea (14%), vomiting (13%), back pain (11%) [54].
Neratinib	Pathological complete response in 56% of HER2-positive but HR- breast cancer patients compared to 33% in the control group. Further, 84% response rate in HER2-positive and hormone receptor-positive compared to a 59% response rate in HER2+ and hormone receptor-negative [55].	Diarrhea (83.9%), nausea (37.9%), abdominal pain (28.4%) [55].
Gefitinib	No complete or partial responses observed in previously treated patients with advanced breast cancer [56].	Diarrhea (45.2%), skin rash (12%) [56].
Afatinib	Partial response in 10% and progressive disease in 39% of extensively pretreated HER2-positive patients metastatic breast cancer progressing after trastuzumab. No complete response observed [57].	Diarrhea (24.4%), skin rash (9.8%) [57].
